# Mr. Whalley, on Vaccine Inoculation

**Published:** 1802-07

**Authors:** D. Whalley

**Affiliations:** Great Fenton, near Newcastle, Staffordshire


### *To the Editors of the Medical and Phyfical Journal.*

GENTLEMEN,

IN your very ufeful work for the laft month, Mr. Knight recommends a plan for collecting the teftimony of thofe of the Medical Profeffion throughout the kingdom who are favourers of, and have practifed and witneffed the beneficial effects of the Vaccine Inoculation. The execution of this plan, I fear, is rather to be hoped than expected.

I believe few perfons are more fully convinced of the great value of the Difcovery than myfelf, or feel a more fincere wifh that the Difcoverer may receive thofe Honours and Rewards, to which the great bleffing he has beftowed on mankind juftly entitle him.

I confider the benefits of the Vaccine Inoculation too apparent, and the teftimony in its favor too complete, to need the further communication of any individual: But as it cannot be made known too publicly, how far and how generally the practice is already extended, I am defirous, through the medium of your Journal, to communicate the refult of my practice.

I began

I began in the autumn of the year 1800, and continued the inoculation through the following winter, in a limited mode, that I might obferve its progrefs, and become acquainted with the appearances of the diforder.

In the fpring of 1801, I offered a general inoculation to the poor of this populous part of the kingdom, which I had the pleafure to find very generally received; I difcontinued the inoculation the three fummer months, and recommenced it in the month of October following, from which time I have uninterruptedly continued it.

I have inoculated above two thoufand perfons; I have feen no puftular eruptions befide the inoculated puftules, excepting a very few cafes where one or two minute ones have appeared, not far from the inoculated one; and I have met with no one circumftance, which has given me a moment's concern, or in the leaft fhaken that confidence in the difeafe which my practice was progreffively eftablifhing in my mind.

Previous to the recommendation of Dr. Jenner, that matter fhould not be taken for inoculation later than the eighth day; that I might be more certain of procuring matter, I ufually examined my patients, and took it on the morning of the tenth day. But fince the fact has been afcertained, that the matter taken fo late as the tenth day, or when the areola has fpread, is lefs active, I have been in the practice of taking matter on the eighth day.

Once only have I been under the neceffity of deviating from this rule; and as it gives another clear proof that a change does take place in the power of the virus, and the inoculation was on rather a large fcale, I will ftate the circumftances.

I had engaged to inoculate the children of the poor in the village of Eggington, in Derbyfhire, and in my way thither I paffed through the village of Marfton, where I had inoculated a few children fome days before, and the poor of Marfton and the neighbourhood requefted I would inoculate their children.

As I thought I had plenty of matter, I appointed the next day, but at Eggington I found affembled a much greater number of children than I expected; I inoculated ninety-three, and found I had expended nearly the whole of the matter I was provided with.

To perform my promife at Marfton, I was obliged to revert to the children from whom I had taken the matter for the Eggington inoculation, and I felected two on whom the pocks had appeared rather backward on the eighth day, and on that account from whom I had taken no matter; but now, on the tenth day, the pocks were fully formed and the efflorefcence fpreading; the matter was abundant, and perfectly fluid and limpid.

With

With this matter, on the fame day it was taken, I inoculated fixty-nine children at Marfton.

When I examined the children at Eggington, only three were uninfected; and with the exception of three or four, the whole fhewed the difeafe in a regular and perfect form. Of the fixty-nine children at Marfton, feventeen were uninfected; and on many of thofe who had taken the difeafe, it appeared in a languid backward ftate on the eighth day.

One child I inoculated again, as I was doubtful whether the fpeck of inflammation which appeared would produce the difeafe; but I found eight days after that it had fucceeded, and the areola appeared on the thirteenth day.

Another I inoculated again, becaufe the pocks were backward, fmall, and acuminate, and wore the character of the Small-pox, rather than the Cow-pock puftules; but the firft inoculation went on progreffively, and the fecond did not take effect. I am, &c.

D. WHALLEY, jun.

*Great Fenton, near Newcastle, Staffordshire, May* 16, 1802.

P.S. Many of my patients were expofed to the fevereft tefts of variolous infection after the Cow-pox, but none of them took the Small-pox.

